# MS-based glycomics: An analytical tool to assess nervous system diseases

**DOI:** 10.3389/fnins.2022.1000179

**Published:** 2022-11-03

**Authors:** Wenjing Peng, Firas Kobeissy, Stefania Mondello, Chloe Barsa, Yehia Mechref

**Affiliations:** ^1^Department of Chemistry and Biochemistry, Texas Tech University, Lubbock, TX, United States; ^2^Program for Neurotrauma, Neuroproteomics and Biomarkers Research, Department of Emergency Medicine, University of Florida, Gainesville, FL, United States; ^3^Department of Biomedical and Dental Sciences and Morphofunctional Imaging, University of Messina, Messina, Italy

**Keywords:** mass spectrometry (MS), glycomics, nervous system diseases, glycan markers, LC-MS/MS

## Abstract

Neurological diseases affect millions of peopleochemistryorldwide and are continuously increasing due to the globe’s aging population. Such diseases affect the nervous system and are characterized by a progressive decline in brain function and progressive cognitive impairment, decreasing the quality of life for those with the disease as well as for their families and loved ones. The increased burden of nervous system diseases demands a deeper insight into the biomolecular mechanisms at work during disease development in order to improve clinical diagnosis and drug design. Recently, evidence has related glycosylation to nervous system diseases. Glycosylation is a vital post-translational modification that mediates many biological functions, and aberrant glycosylation has been associated with a variety of diseases. Thus, the investigation of glycosylation in neurological diseases could provide novel biomarkers and information for disease pathology. During the last decades, many techniques have been developed for facilitation of reliable and efficient glycomic analysis. Among these, mass spectrometry (MS) is considered the most powerful tool for glycan analysis due to its high resolution, high sensitivity, and the ability to acquire adequate structural information for glycan identification. Along with MS, a variety of approaches and strategies are employed to enhance the MS-based identification and quantitation of glycans in neurological samples. Here, we review the advanced glycomic tools used in nervous system disease studies, including separation techniques prior to MS, fragmentation techniques in MS, and corresponding strategies. The glycan markers in common clinical nervous system diseases discovered by utilizing such MS-based glycomic tools are also summarized and discussed.

## Impact of neurological diseases and urgent need for clinical markers

At present, more than 47 million people are suffering from dementia, and this number is expected to exceed 130 million by 2050 as the world’s population ages (Alzheimers Association, 2020). Increased longevity, the result of modern medical and public health advances, dramatically increases the risk of neurodegenerative diseases. Such diseases, affecting the nervous system and characterized by a progressive decline in brain function and progressive cognitive impairment, decrease the quality of life for those afflicted as well as their families and loved ones (Super et al., 2019). In fact, the great majority of these patients are cared for by family members who assist in most, if not all, facets of daily living activities (Nikzad-Terhune et al., 2010). Caregiving for dementia patients has also been shown to negatively affect both the physical health and psychological wellbeing of those providing care. A meta-analysis conducted in 2003 revealed that dementia caregivers have elevated levels of stress hormones which put them at greater risk for developing cardiovascular diseases, hypertension, and type II diabetes (Vitaliano et al., 2003). Moreover, spouses caring for dementia patients have an elevated risk of receiving antidepressant treatment, due to the increased probability of depression affecting them compared to non-caregivers (Richardson et al., 2013). The factors that may influence caregiver burden include the severity of the patient’s dementia, the behavioral disturbances of the patient, and the levels of neuropsychiatric symptoms such as delusions and hallucinations (Etters et al., 2008).

## Glycosylation

### General concepts and association with diseases

Glycans, which are mostly found on the outermost surfaces of cellular and secreted macromolecules, are widely distributed in nature and are highly diverse in both prokaryotes and eukaryotes (Kristic and Lauc, 2017). The importance of glycans is not just restricted to the structural features they convey; in fact, they display regulatory activity and play an important role in a variety of functions in both physiological and pathophysiological states (Varki, 2017). In this section, we discuss the main types of glycosylation, the role that glycans play in some diseases, and the possibility to use them as biomarkers of pathogenesis.

Many of our system’s proteins are modified by the addition, through β-1N linkage, of an N-acetylglucosamine (GlcNAc) to an Asn side chain, a process known as N-glycosylation. This attachment allows for additional modifications such as galactosylation, GlcNAcylation, sialylation, and fucosylation; depending on the nature of the latter modification, the final obtained structure is a high mannose N-glycan, a hybrid N-glycan, or a complex N-glycan (Richard et al., 1983). The synthesis of N-glycans begins in the endoplasmic reticulum where the transfer of the ligand-glycan precursor GlcNAc to Asp is achieved through oligosaccharyltransferase (OST) activity. Then, after sequential removal of the glucose residues and the Man residue by α-glycosidases (α-Glc I-II) and α-mannosidase (ER α-Man), respectively, the glycoprotein will be further modified through trimming by specific mannosidases and/or additional glycan modifications occurring in the Golgi apparatus (Parodi, 2000).

O-glycosylation usually occurs on oxygen atoms of Ser and Thr, amino acids characterized by the presence of functional hydroxyl groups in their structure (Ten Hagen et al., 2001). Classification of the end O-glycans formed depends on the initial sugar that was attached to the protein and on the other sugar structures which are subsequently added; the addition of N-acetyl-galactosamine on Ser or Thr, known as the mucin-type linkage, is the most widespread type of O-glycosylation (Carraway and Hull, 1991). In fact, glycosyltransferases will expand the existing structure that was formed in the endoplasmic reticulum with galactose (Lis and Sharon, 1993), GlcNAc, sialic acid, and fucose (Harris et al., 1991) in the Golgi apparatus. This is similar to the N-glycosylation process, with one important distinction: O-glycans do not undergo trimming like N-glycans.

N-glycosylation and O-glycosylation are not the only two types of glycosylation. The addition of glycosylphosphatidylinositol (GPI) anchors at the carboxyl terminus of some proteins, a process termed glypiation, is omnipresent in eukaryotic organisms (Ferguson, 1992). This type of glycosylation, performed by a transamidase found in the endoplasmic reticulum, has been found to play a role in transmembrane signaling and folate uptake (Ferguson, 1991). However, additional studies are still required to investigate glypiation and C-mannosylation, another lesser-targeted glycosylation, and the effects that such types of glycosylation have on physiological processes.

Research over the years has revealed that genetic defects in glycosylation lead to embryonic lethality, exposing the vital role of glycans, with the identification of more than a hundred congenital disorders of glycosylation (CDGs) resulting from glycogenes’ deficiencies (Haeuptle and Hennet, 2009). Because glycans play an essential role in muscular, developmental, and neurological processes, CDGs are typically severe in their manifestations. Hence, studying glycans as biomarkers of certain diseases seems like a promising tool that has interested many scientists in the field.

The most common form of CDG is the N-glycan-related PMM2-CDG, with phosphomanomutase 2 (PMM2) being responsible for the conversion of Man-6-P to Man-1-P. Because Man-1-P is essential for the production of the precursor of an N-glycan, this CDG presents with a neurological or multi-system phenotype depending on the type of mutation affecting the gene (Witters et al., 2019). O-glycan-related CDGs have been associated with the development of congenital muscular dystrophies as a result of abnormal Man O-glycosylation of the alpha-dystroglycan protein. The six genes that were identified to cause muscular dystrophy (POMT1, POMT2, POMGnT1, FCMD, FKRP, and LARGE) are all associated with an under-glycosylation of α-dystroglycan (Martin, 2007). Also, the overexpression of the LARGE gene increases dystroglycan glycosylation and allows the restoration of its function (Barresi et al., 2004). This confirms the important role that O-glycosylation plays in avoiding the development of such a disease.

### The successful story of glyco-biomarkers in other diseases

Because glycosylation is greatly affected by inflammation and diseases such as cancer and diabetes, the use of glycans as biomarkers in the study of diseases is a promising tool. The synthesis of glycans has been shown to be more affected by disease states than protein synthesis, and quantifying oligosaccharide expression is easier than investigating protein expression, which supports the use of glycans as biomarkers.

Glycosylation biomarkers are already in use in the cancer field as a means to identify cells with stem-cell-like phenotypes. In fact, abnormal core fucosylation and increased N-glycan branching are two common features that are observed in cancer cells (Pinho and Reis, 2015). Also, elevated α-2,6-linked sialic acid in N-glycans seems to be a characteristic of pancreatic and colon cancers and is used as a glycosylation-related biomarker (Wilson and Massey, 1987).

Moreover, alterations in the glycosylation of IgG accompanied by an elevated amount of bisecting GlcNAc and core fucose on the antigen-binding fragment (Fab)-associated glycans seem to be a critical component of rheumatoid arthritis pathogenesis (Youings et al., 1996). Abnormal O-linked N-acetylglucosamine-mediated signaling and enhanced glycation of multiple proteins such as AKT (Liu et al., 2000) seem to be glycosylation-biomarkers of diabetes mellitus.

Many proteomics studies have been conducted to identify biomarkers of neurodegenerative disorders (Zhang et al., 2005; Shi et al., 2009, 2015; Vanguilder and Freeman, 2011), yet few reports are to be found on the CSF N-glycoproteome study. Considering that many neurodegenerative disorders are associated with alterations in glycosylation patterns (Hwang et al., 2010), and taking into account the success that glycosylation-based biomarker discovery has achieved in other fields (and especially in cancer research), discovering glycan markers for dementia-related diseases seems promising for the diagnosis, prognosis, and even treatment of such burdensome diseases (Chen et al., 2010a; Ahmed et al., 2014). Some of the main findings from diverse *in vitro* and *in vivo* studies revealing the important role that glycosylation plays in the development of neurodegenerative disorders are summarized in Table 1.

**TABLE 1:** Summary of glycoproteins, corresponding pathologies resulting from glycosylation modifications, and their suggested underlying mechanisms.

Glycoprotein/ Glycomarker	Glycosylation type	Associated disease	Source	Underlying mechanism	References
APP	Bisecting N-GlcNA	Alzheimers meisease	CSF, blood plasma and saliva	Aß accumulation, inflammation, oxidative stress and mitochondrial impairment	Hwang et al., 2010
BACE1	Bisecting N-GlcNA	Alzheimer010pisease	CSF	Aß accumulation, impaired neuronal function	Vanoni et al., 2008; Kizuka et al., 2015
Nicastrin	O-glycosylation	Alzheimertionisease	CSF	Decreased γ-secretase activity and Aß production	Yang et al., 2002
Tau	N-glycosylation O-glycosylation	Alzheimertionisease	CSF and blood plasma	Microtubule disruption, formation of neurofibrillary tangles	Liu et al., 2002; Losev et al., 2021
Acetylcholinesterase (AChE)	N-glycosylation	Alzheimertionisease	CSF and brain tissue	Increase in amyloid, stress, and inflammation	Appleyard and McDonald, 1992; Saez-Valero et al., 1999
Released N-glycans	N-glycosylation	Alzheimertionisease	Brain tissue	Decrease of bisecting and core-fucosylated N-glycans	Gizaw et al., 2016
*In situ* N-glycan MALDI-imaging	N-glycosylation	Alzheimertionisease	Brain tissue	Hyper-N-glycosylation in the frontal cortex regions	Hawkinson et al., 2022
α-synuclein	O-GlcNAcylated	Parkinsontedgisease	CSF, brain tissue and blood plasma	Synaptic disruption	Vicente Miranda et al., 2017; Levine et al., 2019
TREM2	N-glycosylation	Parkinsontionisease	Blood plasma	Oxidative stress and inflammation	Rayaprolu et al., 2013
DJ-1	–	Parkinson013disease	CSF	Oxidative stress	Advedissian et al., 2016; Sharma et al., 2019
Parkin	–	Parkinson)19risease	CSF	Neurotransmission disruption	Chung et al., 2001; Shimura et al., 2001
Narp (NPTXII)	N-glycosylation	Parkinsontionisease	CSF	Synaptic disruption, neurotransmission impairment, cell death	Moran et al., 2008
Released O-glycans	O-glycosylation	Parkinsontionisease	Brain tissue	Increase of sialylation	Wilkinson et al., 2021

## Mass spectrometry-based separation techniques facilitate glycomics studies in neurological diseases

MS has become a common tool in glycomics analysis due to its high resolution and ability to acquire rich structural information. However, MS alone sometimes faces sensitivity issues due to the competitive ionization that hinders the characterization of low abundant glycans. Therefore, numerous separation techniques have been widely coupled to MS to reveal glycan microheterogeneity and biological functions. Of these techniques, the most common include pre-ionization separations such as reverse phase chromatography (RPLC), hydrophilic interaction chromatography (HILIC), and capillary electrophoresis (CE), as well as post-ionization separations such as ion mobility (IM). In pre-ionization separation techniques, any ionization competition is addressed since glycans are separated and ionized separately; in post-ionization separation techniques, however, this issue still requires consideration. Although separation techniques offer advantages in glycomics, MS-based strategies such as matrix assisted laser desorption/ionization (MALDI) are still common for clinical glycomics analysis because of the simple sample preparation and high throughput.

### Matrix assisted laser desorption/ionization-mass spectrometry in neurological glycomics

MALDI-MS is one of the most common ways to study glycomic alterations in biological samples (Harvey, 1999), and improving sensitivity is of great importance in MALDI-MS. During the last few decades, several MALDI matrices have been developed to achieve an efficient glycomic analysis (Stahl et al., 1991, 1994; Karas et al., 1993; Metzger et al., 1994; Mohr et al., 1995; Mechref and Novotny, 1998; Jiao et al., 2015), including dihydroxybenzoic acid (DHB) (Jensen et al., 2013; Inyoung et al., 2016; Chen et al., 2020), and nanomaterials such as glutathione-capped iron oxide and carbon nanoparticles that have recently been used as a co-matrix to enhance sensitivity (Liang et al., 2014; Banazadeh et al., 2018).

#### Matrix assisted laser desorption/ionization-mass spectrometry

MALDI is one of the soft ionization techniques that enable less in-source fragmentation for an efficient structural elucidation. MALDI has successfully profiled glycans in many diseases such as liver cancer (Kamiyama et al., 2013; Kim et al., 2015), breast cancer (Kyselova et al., 2008; de Leoz et al., 2011), ovarian cancer (Biskup et al., 2013, 2017), colorectal cancer (Kaprio et al., 2015; Liu et al., 2018), and more (Qin et al., 2017; Wang et al., 2018). Time of flight (TOF) MS is often coupled to MALDI for its fast scan speed and theoretically unlimited *m/z* range, which together enhance the efficiency in analysis of large biological cohorts (Everest-Dass et al., 2018). However, the sensitivity remains a challenge even with the introduction of a different matrix and co-matrix. Therefore, derivatization strategies, such as Girard’s reagent P/T (Kim et al., 2015; Zhang et al., 2019, 2020a) and permethylation (Kang et al., 2005; Shubhakar et al., 2016), have been used to improve the ionization efficiency. In addition, sialic acid derivatization methods, such as amidation (de Haan et al., 2015), esterification (Gomes de Oliveira et al., 2015; Powers et al., 2015), and alkylamidation (Nishikaze et al., 2017; Hanamatsu et al., 2018; Suzuki et al., 2019), were used to distinguish sialic acid linkage isomers and stabilize labile sialic acid structures. Among these, sialic acid linkage specific alkylamidation (SALSA) is the newest efficient and stable linkage specific derivatization method (Nishikaze et al., 2017). The following methods were mainly modifications based on SALSA. With the demonstration of these MALDI-MS strategies in many diseases, MALDI-MS has also been widely applied in glycomic analysis of central system diseases.

Early in 2013, MALDI-TOF/TOF (Applied Biosystems 4800) was applied to profile the permethylated glycans derived from congenital disorders of glycosylation (CDGs) patient sera and proved to be able to differentiate CDG from controls (Xia et al., 2013). Recently, Duvet et al. (2018) investigated the serum N-glycome expressions in MAN1B1-CDG patients using the same MAIDI-TOF/TOF system in combination with an Endo-β-N-acetylglucosaminidase H (Endo H) digestion. Since the Endo H can only release high-mannose and hybrid structures, a straightforward investigation of MAN1B1 deficit was achieved. The MALDI-MS successfully identified the accumulation of several hybrid structures and alteration of high-mannose structures in MAN1B1-CDG patients.

Gizaw et al. (2016) used a glycoblotting method (BlotGlyco^®^ H beads, Sumitomo Bakelite, Tokyo, Japan) to enrich released N-glycans followed by an on-beads methyl esterification to investigate the glycan expression changes in AD patients’ brain tissue using MALDI-TOF-MS. This analytical method allowed the identification of 72 N-glycan compositions from brain tissues of AD patients. The decrease of bisecting and core-fucosylated N-glycans in the frontal cortex of AD patients was observed, which could be explained by the downregulation of glycosyltransferases GnTIII and FUT8 in the brain. This method was also demonstrated on AD serum and CSF N-glycomics.

Recently, Palmigiano et al. (2018) introduced a MALDI-TOF/TOF (Applied Biosystems 4800) based method for the analysis of permethylated glycans in Alzheimer’s Disease (AD), which allowed the identification of 90 N-glycan structures. Later, permethylated glycan profiling using a 4800 MALDI-TOF/TOF mass spectrometer (PerSeptive Biosystems) revealed an increase in the levels of bisecting Lewis × epitopes in certain glycans (Schedin-Weiss et al., 2020). Besides CDG and AD, MALDI-TOF-MS was also employed in glycomics analysis of other neurological diseases such as amyotrophic lateral sclerosis (ALS) (Edri-Brami et al., 2012; Costa et al., 2019) and Huntington’s Disease (HD) (Gizaw et al., 2015).

Although MALDI-MS has been applied in many neurological glycomics studies, the technique’s sensitivity was limited. Compared to other methods, there were usually fewer glycan structures identified in MALDI-MS (Huffman et al., 2014). However, because of the high throughput and simple sample preparation, it is promising to couple MALDI-MS with *in situ* glycomics techniques such as mass spectrometry imaging (MSI).

#### Matrix assisted laser desorption/ionization imaging

In the last decade, a major application of MALDI-MS has been MALDI-MSI, which can visualize the *in situ* distributions of different glycans on a tissue slide. The key factors in MALDI-MSI are the raster-scan resolution and the MS resolution and accuracy. The scan resolution determines the spot (pixel) number measured by MS (with a spatial resolution down to 5–20 μm), while the MS resolution and accuracy determines the molecules that can be analyzed (Aichler and Walch, 2015). It enables a label-free *in situ* characterization of hundreds of glycans on a single tissue section (Hasan et al., 2021). The most common MS techniques employed in MALDI-MSI are FTICR for high-resolution analysis or TOF for rapid and high mass range analysis. MALDI-MSI has been used to investigate glycans from neurological tissue samples for years.

The MALDI-MSI analysis of N-glycans on a brain tissue was first introduced by Powers et al. (2013). The typical *in situ* N-glycomic imaging protocol has also been summarized (Drake et al., 2018; Stanback et al., 2021). Toghi Eshghi et al. (2014) utilized this strategy to analyze the N-glycan distributions in a glioblastoma tumor transplanted mouse brain using a MALDI-QIT-TOF MS (AXIMA Resonance, Shimadzu). In this study, a 100 μm imaging resolution was employed, resulting in identification of 42 N-glycans. The different distributions of several glycans in tumor vs. normal brain tissues were identified. In another study, Malaker et al. (2022) employed a RapiFlex MALDI-TOF MS (Bruker) together with a MALDI LTQ orbitrap XL (Thermo Fisher Scientific) to map the N-glycan distribution of canine glioma samples, and identified the increase of a bi-sialylated bi-antennary structure in canine glioma.

**FIGURE 1:**
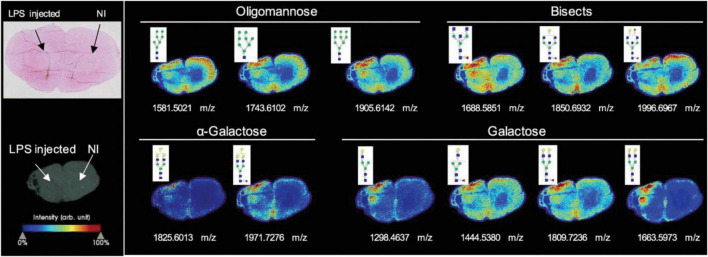
N-glycan imaging of the brain of LPS-injected rats. Main N-glycan structures that are visualized in the lesion core belong to specific groups: oligomannose, bisected structures, and galactosylated glycans, mainly with α-galactose. Each image is accompanied by the putative structures determined by combinations of accurate m/z, CID fragmentation patterns, and glycan database structure. NI stands for none injected area. Reprinted with the permission from Rebelo et al. (2021).

Most recently, Rebelo et al. (2021) investigated the glycosylation changes during neuroinflammation in the rodent brain. The rat striatum was injected by lipopolysaccharide (LPS) to establish the striatal neuroinflammation model. The N-glycan imaging was performed using a MALDI timsTOF fleX trapped ion mobility QTOF MS (Bruker) with a laser spot of 20 μm and a raster width of 40 μm between spots. Overall, 52 N-glycans were detected by MALDI-MSI. Some significant glycan expression changes were observed in the injected site that were directly related to the inflammatory reaction, as shown in Figure 1. These significant N-glycans were mainly highlighted by the increase of high-mannose, bisecting, and α-galactosylated glycans in the lesion core. Meanwhile, some fucosylated and sialylated structures were observed to have decreased in the injected side. Later, Hawkinson et al. (2022) spatially investigated N-glycan heterogeneity in both mouse and human AD brain samples and corresponding age-related controls using an optimized *in situ* MALDI-MSI workflow. The imaging was acquired using a Waters SynaptG2-Xs MS coupled to traveling wave ion mobility. The spatially heterogeneous N-glycans were identified as a potential AD clinical hallmark, and the hyperglycosylation as a phenotype of AD mouse models and human frontal cortex specimens were discovered. These studies demonstrate the potential for glyco-marker discovery with MALDI-MSI. However, the majority of the studies were on animal models or limited biomedical samples. An increased biological cohort should be considered in future works.

A new laser-induced postionization technique (MALDI-2) has been introduced to MALDI-MSI to enhance the sensitivity through a wavelength-tunable postionization laser in the gas phase of ionized plume (Soltwisch et al., 2015). This technique initializes a secondary MALDI-like ionization process to increase the signal intensity and was successfully utilized on brain tissue glycome imaging, which enhanced the sensitivity by 10 times relative to the current standard method (Heijs et al., 2020). Although MALDI-2 imaging has not yet been used in glycomics analysis of nervous system diseases, the greater sensitivity makes it a promising alternative in future studies.

Although quantitative MSI (qMSI) of metabolites and peptides have been illustrated as a result of significant advances in MSI (Tobias and Hummon, 2020), qMSI of glycans is still a challenging analytical task. Generally, qMSI has been difficult to attain due to matrix crystallization heterogeneity, tissue heterogeneity, insufficient analyte extraction, and ion suppression effect. Ellis et al. (2014), Rzagalinski and Volmer (2017), and more recently Tobias and Hummon (2020) have highlighted the challenges of qMSI for drugs, lipids and peptides. qMSI of glycan is achieved by attending to the same sources of variability that are commonly associated with MALDI-MS measurement, including ion suppression, sample heterogeneity, spot-to-spot variability, sample preparation, matrix choice, matrix application and analyte recovery.

Like any analytical measurement, qMSI requires multiple replicates. Multiple tissue sections should be mounted on the same target plate to allow multiple measurements of the same biological or chemical conditions. Replicate analyses are critical to minimize any potential measurements variabilities originating from sample treatment or matrix deposition. Triplicates are also required to construct calibration curves. Biological replicates should be also considered when constructing calibration curves. When possible, a minimum of three biological replicates should be considered to ensure that the observed variation is due to the condition under investigation and not because of random variation (Tobias and Hummon, 2020).

Analytical sensitivity in qMSI is directly dependent on sample preparation and correct choice of matrix. Sample preparation such as washing is critical to ensure the removal of any materials that could interfere with the proper crystallization of the matrix or the suppression of the MS signal of the analytes. Several studies have detailed sample preparation for the MSI of proteins (Groseclose et al., 2007; Seeley and Caprioli, 2008; Heijs et al., 2016; Zubair et al., 2016) and gangliosides (Angel et al., 2012; Yang et al., 2019). The choice of matrix in qMSI is driven by sensitivity, MS-mode of analysis and suitability of the matrix for the lengthy time of analysis commonly associate with MSI. Also, the use of robotic sprayer is highly recommended to ensure uniform application of matrix and internal standards (needed for absolute quantification). Thus far, no studies have addressed sample preparation needed for MSI of glycans; however, several of the steps discussed for the sample preparation of biomolecules are applicable to glycan MSI. Glycan MSI has been mainly conducted using α-cyano-4-hydroxycinnamic acid (CHCA) as a matrix (Shi et al., 2019).

Defining a quality control (QC) sample is an excellent mean to address sources of variability in MSI; however, identifying such a QC sample might not be an easy task especially for qMSI glycomics. Recently, the mean signal intensities of peptides associated with egg white section as a QC sample has been used to monitor the performance of a large scale formalin-fixed paraffin embedded formalin-fixed paraffin embedded (FFPE) MALDI-MSI study using a TOF/TOF instrument (Condina et al., 2019). Also, mimetic tissue model spiked with internal standard has been used to monitor the performance of qMSI in multicenter study (Barry et al., 2019).

The same guidelines considered for absolute quantification in LC-MS and GC-MS are applied to qMSI of peptides and metabolites (Tobias and Hummon, 2020). Several calibrants should be considered when constructing the calibration curve. The calibration points should be evenly distributed in the linear working range of the curve. The signal should be averaged over the region of interest (ROI). The signals of the different adducts formed should be plotted against the applied concentration (Tobias and Hummon, 2020). The signal of the analyte generated from the real samples can be then determined using the constructed calibration curve. Although no studies thus far have highlighted absolute quantitation of glycan through qMSI, the steps summarized up for other molecules can be utilized. Cambridge Isotope Laboratories Inc. (Tewksbury, MA) is offering several isotopically labeled glycans that could be potentially used as both internal standards and calibrants for absolute qMSI of glycans. GlycoScientific, LLC (Athens, GA) is offering iGlycoMab which is a murine IgG2b monoclonal antibody whose glycans are labeled with the stable isotope (15N).

#### Matrix assisted laser desorption/ionization-mass spectrometry based isomeric glycomics

Without separation, isomeric characterization becomes a challenge for MALDI-MS. Thus, linkage specific derivatization methods have been widely applied in MALDI-MSI to distinguish *in situ* sialic acid linkages. Differential derivatization, including amidation (de Haan et al., 2015) and esterification (Gomes de Oliveira et al., 2015; Powers et al., 2015), can introduce different mass shift of salic acids with different linkages. These mass differences can be differentiated by MALDI-MS, thus achieving the characterization of sialic acid linkage isomers. The earliest derivatization method was introduced by Wheeler et al. (2009), then improved by Reiding et al. (2014). Later, a two-step derivatization method was developed by Holst et al. (2016) and further modified as the sialic acid linkage specific alkylamidation (SALSA) method (Nishikaze et al., 2017; Hanamatsu et al., 2018; Suzuki et al., 2019) to increase the labeling efficiency; this has been used with the isotopic labeling technique (iSALSA) in liver disease using MALDI-TOF to characterize sialylated glycan isomers (Hanamatsu et al., 2019). Recently, an enzymatic method to distinguish core- and branch-fucosylation was introduced (West et al., 2020), where the endoglycosidase F3 (Endo F3) was utilized to introduce 349.137 Da mass shift between Endo F3 digested and PNGase F digested core-fucosylated N*-*glycans. These derivatization methods have been employed in MALDI-MSI for *in situ* isomeric glycomics of multiple diseases (Powers et al., 2013; Holst et al., 2016; Herrera et al., 2019; Blaschke et al., 2020; West et al., 2020). However, isomeric differential derivatization methods have not been widely applied in CNS diseases. The fact that glycan isomers have been demonstrated to be involved in many diseases prompts a need to use these methods in MALDI-MS-based isomeric glycomics in future studies.

Although MALDI-MS and MALDI-MSI provide rapid analytical speed, high throughput, and ability of visualization, the relatively low resolution and sensitivity hinders its application in analyzing low abundant glycans from precious biomedical samples. Therefore, different separation techniques are commonly employed prior to MS to achieve better sensitivity and selectivity of glycans.

### Liquid chromatography-mass spectrometry in neurological glycomics

Among the many separation techniques, liquid chromatography (LC) is the most widely used approach in glycomic analysis due to its high reproducibility, high sensitivity, and compatibility with MS. However, the relatively low ionization efficiency of glycans remains a challenge in glycomic analysis. To overcome this issue, a series of derivatization methods were developed to enhance ionization and separation. One of the common derivatizations is the conjugation of different reagents on the reducing end of glycans. The reactions with the glycan reducing end can be (i) reductive amination such as 2-aminobenzoic acid (2-AA) (Ruhaak et al., 2008), 2-aminobenzamide (2-AB) (Royle et al., 2008), procainamide (ProA) (Klapoetke et al., 2010; Kozak et al., 2015), 2-aminopyridine (PA) (Deguchi et al., 2008), 2-aminonaphthalene trisulfonic acid (ANTS) (Lemoine et al., 2000), and 1-aminopyrene-3,6,8-trisulfonic acid (APTS) (Laroy et al., 2006); (ii) hydrazide chemistry such as Girard’s T reagent (GT) (Gil et al., 2008; Unterieser and Mischnick, 2011), Girard’s P reagent (GP) (Kim et al., 2015), phenylhydrazine (Lattova and Perreault, 2003), and 4-phenethylbenzohydrazide (phenyl2-GPN) (Walker et al., 2011); and (iii) carbamate derivatization such as RapiFluor-MS (RFMS) (Lauber et al., 2015) and InstantPC (Xie and Butler, 2022). Another common derivatization technique of glycans is permethylation, where active hydrogens on hydroxyl and amide groups are replaced by a methyl group. Permethylation provides advantages to stabilize labile sialic acids, prevent fucose migration, and significantly increase sensitivity. Zhou et al. (2017c) compared the most common derivatization techniques including 2-AB, ProA, aminoxyTMT, RMFS, and permethylation. RFMS obtained the highest signal for neutral glycans but distorted the sialylated glycan quantitation, while permethylation gained sensitivity for both neutral and sialylated glycans and is accurate in sialylated glycan quantitation. Different derivatization strategies are employed according to different research goals and separation methods used in the studies. These labeling reagents are important to glycomic studies, since most used one of these reagents to enhance sensitivity, improve separation, or even introduce UV absorption.

#### Liquid chromatography-mass spectrometry analysis of native and reducing end-labeled glycans

Native and reducing end-labeled glycans are highly hydrophilic. A normal reverse phase column (such as a C18 column) cannot be efficient in separation, because reverse phase chromatography relies on hydrophobic interactions. Therefore, hydrophilic interaction chromatography (HILIC) has been widely used in the separation of native and reducing end-labeled glycans. In addition, a porous graphitic carbon (PGC) column is also used in native glycomics due to its polar interactions.

##### Hydrophilic interaction chromatography in neurological glycomics

HILIC-MS has been a popular choice to separate native (Wuhrer et al., 2004) or reducing end-labeled glycans (Wuhrer et al., 2004; Clarke et al., 2009). Different glycan structures can be resolved by their hydrophilicities. Therefore, the gradient used for a HILIC column is usually from high organic phase (e.g., 80% ACN in water) to high aqueous phase (e.g., 20% ACN in water) with the existence of additives such as ammonium formate (e.g., 50 mM ammonium formate in mobile phase A). HILIC-MS has been demonstrated for glycomic analysis with a variety of diseases (Abd Hamid et al., 2008; Arnold et al., 2011; Doherty et al., 2018; Terkelsen et al., 2018), thus proving its potential application in neurological diseases.

In a study of glycomic changes in aging brain nigrostriatal pathways, an in-house packed amide-80 (Tosoh Biosciences) column and glycanpac AXH-1 (Thermo Fisher Scientific) column were coupled to an LTQ Orbitrap XL MS to analyze the N-glycans and Heparan Sulfate (HS) disaccharides, respectively (Raghunathan et al., 2018). The age-related changes of nigrostriatal N-glycosylation and HS disaccharides were observed, which might provide a deeper insight to improve the viral vectors’ design for Parkinson Disease (PD). Another study of glycomics in aging and PD also employed an in-house packed nano HILIC column interfaced with an Orbitrap XL MS (Thermo Fisher Scientific) to analyze HS disaccharides (Raghunathan et al., 2020). The changes of HS and chondroitin sulfate (CS) domain structures were identified in aged and PD patients’ brains. Noticeably, this study reported the capability of HILIC in separating disaccharide isomers. These recent examples demonstrate the capability of HILIC-MS for glycomic analysis of neurological diseases, and have been applied in additional studies (Russell et al., 2017; Samal et al., 2020).

**FIGURE 2:**
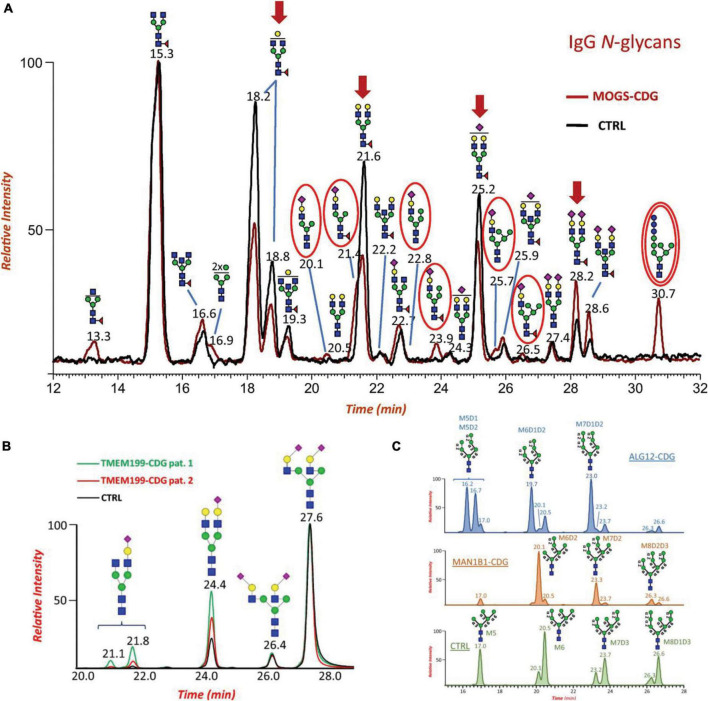
**(A)** Overlaid Total Ion Current Chromatograms (TICCs) of RapiFluor-MS labeled IgG N-glycans from a patient with MOGS-CDG (red line) and control (black line). Major changes of specific glycoforms are noted in patient profile by red arrows. The presence of disease-specific oligomannose N-glycan biomarkers (Glc_3_Man_7_GlcNAc_2_) is highlighted with a double red circle. **(B)** Extracted ion chromatograms (EICs) of major RapiFluor-MS labeled biantennary and sialylated N-glycans identified in serum of patient 1 with TMEM199-CDG (green line), patient 2 with TMEM199-CDG (red line) and control (black line). Disialo-isomers eluted at RT 27.6 and 26.4 min depending on NeuAc linkage positions, monosialo-biantennary species eluted at RT 24.4 min whereas monogalacto- monosialo- isomers, if present, eluted at two different retention times due to differential Gal-NeuAc elongation at the two branches, respectively. **(C)** Comparison of the extracted ion chromatograms (EICs) of the RapiFluor-MS labeled oligomannose serum N-glycans Man_5 – 9_GlcNAc_3_ from a patient with ALG12-CDG (blue chromatogram), a patient with MAN1B1-CDG (orange chromatogram), and a healthy control (green chromatogram). Dotted lines refer to linkages that should be missing in the final structure. Reproduced with the modification and permissions from Messina et al. (2021).

Along with glycan profiling, HILIC can provide isomeric separations of native and reducing end-labeled glycans (Ahn et al., 2010; Moravcová et al., 2021). The differences in the surface hydrophilicities of glycan isomers can be the main force to resolve glycan isomers. Recently, several studies have reported isomeric separations using HILIC-MS in glycomic analysis of diseases associated with nervous systems. Messina et al. (2021) proved the isomeric separation of N-glycans in the study of CDG glycomics, which is considered to be closely related to many neural system diseases. The glycans were labeled by RFMS and analyzed using a HILIC column (ACQUITY UPLC Glycan BEH Amide, Waters) coupled to an Exactive Orbitrap MS (Thermo Fisher Scientific). Figure 2 depicts the separations of N-glycans on the HILIC column. The separation of glycan compositions was achieved as shown in Figure 2A, demonstrating the potential ability of HILIC-MS for neurological glycomics analysis. Additionally, isomeric separations for both sialylated glycans and high-mannose glycans were observed as shown in Figures 2B,C, respectively. The isomeric structures were assigned based on retention times and comparison to MS databases and literatures. With the separation of isomers, distinct isomeric distribution changes could be observed among different CDG patients and controls.

Not only N-glycan isomers, but also O-glycan isomers can be characterized by HILIC-MS. In a recent study of PD glycomics, Wilkinson et al. (2021) investigated the O-glycan expression of striatum and substantia nigra tissue in human PD. The O-glycans were labeled with 2-AB and analyzed by a FLR-HILIC-UPLC-MS (BEH glycan column, Waters; Waters Xevo G2 QTof MS). Baseline separations of both sialic linkage isomers and positional isomers were achieved. The isomeric structures were assigned by comparing retention time, MS and MS^2^ data between FLR-HILIC-UPLC-MS and PGC-LC-MS/MS with the assistance of exoglycosidase array digestion. This approach allowed the identification of 70 O-glycans overall, and revealed a significant increase of sialylation in PD. Although the aforementioned works have proved the potential of HILIC-MS in isomeric glycomic studies, there are fewer studies of neurological diseases relative to other diseases. In addition, larger biological cohorts of neurological diseases need to be further investigated using HILIC-MS.

##### Porous graphitic carbon in neurological glycomics

Besides HILIC, PGC is another separation technique that is widely applied in native and reducing end-labeled glycomic analysis. There are two major forces, hydrophobic interaction and a distinctive polar retention effect, that drive the separation of glycans on PGC, which makes it a dominant material for efficient isomeric separation and used in multiple diseases (Pereira, 2008; Smilowitz et al., 2013; Stavenhagen et al., 2015; Vitiazeva et al., 2015). PGC-LC-MS has been successfully used in glycomic analysis (Hua et al., 2011), and efforts to establish a PGC-MS glycan database have been recently conducted (Aldredge et al., 2012; Abrahams et al., 2018). Li et al. (2020) summarized the typical PGC-LC-MS protocols for native glycans derived from cells and tissues. Ashwood et al. (2019) introduced a glucose unit (GU) assisted standardization of PGC-LC-MS based glycomics. These data ensure the possibility of using PGC-LC-MS in neurological glycomic analysis.

**FIGURE 3:**
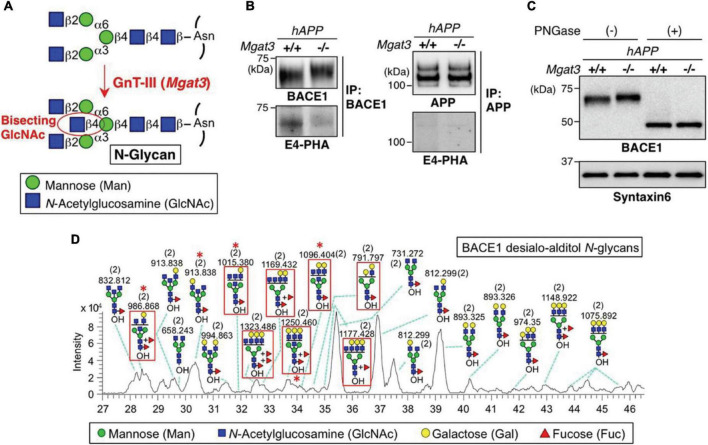
BACE1 is modified with bisecting GlcNAc *in vivo*. **(A)** Bisecting GlcNAc modification by GnT-III. **(B)** BACE1 or APP was immunoprecipitated from mouse brains and blotted with E4-PHA lectin **(Lower)** or anti-BACE1 or APP antibodies **(Upper)**. hAPP indicates the APP23 transgenic mouse model for AD. **(C)** Proteins from mouse brain membrane fractions were treated with or without PNGase F and then immunoblotted for BACE1 or for syntaxin 6 (loading control). **(D)** LC–MS base peak chromatogram of desialo-alditol N-glycans derived from mouse brain BACE1. To simplify the results, N-glycans were chemically desialylated before LC–MS analysis. BACE1-specific glycans, judged by comparison with N-glycan structures from anti-BACE1 IgG, are highlighted by red squares. Asterisks indicate glycans demonstrated by MS/MS analysis to contain a bisecting GlcNAc structure. Numbers in parentheses indicate the charge state. Reprinted with the permissions from Kizuka et al. (2015).

In an early study, PGC (Hypercarb, Thermo Fisher Scientific) –LC-MS (LTQ Orbitrap XL, Thermo Fisher Scientific) was employed to analyze the native desialylated N-glycans derived from the immunoprecipitation pulled β-site amyloid precursor protein cleaving enzyme-1 (BACE1) (Kizuka et al., 2015). Figure 3 depicts the analysis of released N-glycans from BACE1. While the bisecting biosynthesis, immunoprecipitation, and PNGase F digestion of pulled down BACE1 are shown in Figures 3A–C, respectively, the separation of N-glycans on PGC column are exhibited in Figure 3D as a base peak chromatograph with the assignment of major glycan structures. The red asterisks denote the bisecting structures that were identified by the diagnostic ions in MS^2^. Efficient isomeric separation of both positional isomers and linkage isomers were achieved, demonstrating the promising application of PGC-LC-MS in native glycomic studies. An increased bisecting glycan level was observed in AD patients and could be the cause of the accumulation of β-amyloid in AD. Later, the same platform was used to further study the role of bisecting glycans on BACE1, and highlighted its positive effect for BACE1 cleavage (Kizuka et al., 2016).

Recently, Lee et al. (2020) investigated the spatial and temporal N-glycome expressions in human and mouse brains using a nano PGC-chip-LC (Agilent) coupled to an Agilent 6530 Q-TOF MS. The native glycomic analysis resulted in a total of 146 and 130 glycans identified in human and mouse brains, respectively. In addition, 37 isomers corresponding to 23 compositions were characterized by this approach. By comparing the glycan abundances among different brain regions, and between human and mouse, significant alterations of glycosylation were found during postnatal development, while changes of sialylation and fucosylation were observed during prefrontal cortex development. These studies highlighted the role of PGC-LC-MS for native glycan analysis in neurological glycomics.

The use of synthesized glycan isomers as internal standards is another efficient way to identify glycan isomers with high confidence. In an early study, Pabst et al. (2007) introduced the strategy of using biosynthesized glycan isomer standards to assist the accurate identification of PGC-LC-MS based fibrinogen and fibrin glycan isomers derived from different species. Recently, from the same group, Helm et al. (2021) performed PGC-LC-MS to analyze glycans derived from animal and human brains using a glycan retention Time Grid (glyco-TiGr) that contained biosynthesized glycan standards with characteristic mass labels to normalize retention time shift among different runs. This study confirmed that the majority of structures in human brain were the bisected type. Notably, for the first time, the authors reported the identification of hybrid-type glycans with galactosylated and even Lewis X containing bisected N-acetylglucosamine discovered in a natural source. Later, a similar strategy was performed with the use of 136 biosynthesized glycan isomer standards to unambiguously identify corresponding glycan isomers from human brain (Helm et al., 2022). A structure with a bisecting sialyl-lactose and structures with fucose and N-acetylgalactosamine on the same arm were identified in human brain.

#### Liquid chromatography-mass spectrometry analysis of permethylated glycans

Permethylation is one of the most common derivatization strategies in glycomic studies, as it can significantly enhance sensitivity by increasing ionization efficiency, stabilizing labile glycan structures such as sialic acid, and preventing fucose migration during MS/MS (Zhou et al., 2017c). In addition, permethylation provides more information for structural elucidation in MS*^n^*. After permethylation, glycans change from hydrophilic to hydrophobic, thus allowing separation on the most popular column in HPLC, the C18 column. Along with reverse phase chromatography such as C18, PGC is also a promising choice for separating permethylated glycans.

##### C18 in neurological glycomics

Reverse phase (RP) HPLC has been widely used in a variety of fields such as pharmaceutical, life science, industrial food, medical and biomedical, environmental, and more (Žuvela et al., 2019). Among RP materials, the C18 column is the most common and has been dominantly used in chemistry. C18-LC-MS has demonstrated efficient and reliable permethylated glycan profiling for years in many studies (Alley et al., 2012; Hu and Mechref, 2012; Mechref et al., 2012; Hu et al., 2013), with improved separation of permethylated glycans below 55^°^C (Zhou et al., 2016). Although C18-LC-MS is promising in glycan profiling, only partial isomeric separation was observed on a 15 cm C18 column (Thermo Fisher Scientific) (Hanisch and Muller, 2009). A glucose unit index (GUI) standardization was also recently estimated for permethylated glycan analysis using C18-LC-MS (Gautam et al., 2020).

In neurological glycomic studies, C18-LC-MS has been used to investigate glycan expressions in different CNS diseases. A nanoC18-LC-MS system equipped with an Acclaim^®^ PepMap capillary column (Thermo Fisher Scientific) and a LTQ Orbitrap Velos MS (Thermo Fisher Scientific) was employed in a glioma study (Wildburger et al., 2015). Permethylated N-glycans released from brain tissue sections of attractor and non-attractor glioma stem cell xenograft (GSCXs) were analyzed, allowing the identification of 9 N-glycans whose expressions were altered between the two phenotypes. Although on-tissue N-glycan release in this study might lose analytical depth of the intratumoral microenvironment due to the thin slice of tissue, it demonstrated the success of C18-LC-MS in neurological glycomic analysis. In another study, the same C18-LC-MS system was used to investigate the permethylated N-glycan profiles in neuroblastoma cell lines (Hu et al., 2015).

**FIGURE 4:**
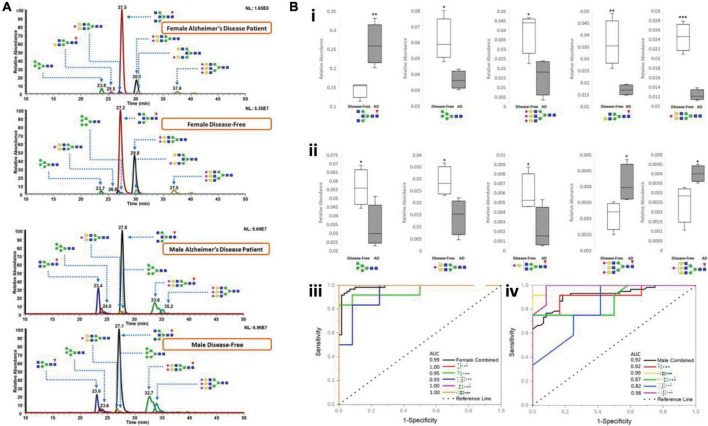
**(A)** Representative chromatograms of various N-glycans, comparing N-glycan expression in CSF between female AD patients and female disease-free, and between male AD patients and male disease-free. **(B)** Box and ROC plots depict the comparison between relative abundances of N-glycan structures in female CSF **(i)** and in male CSF **(ii)** that are statistically significant among the abundant structures. Solids, disease-free; stripes, Alzheimer’s disease. ROC curves for female **(iii)** and male **(iv)**. Some of the plots are hidden due to overlapping. Blue square, N-acetylglucosamine (GluNAc); yellow square, N-acetylgalactosamine (GalNAc); green circle, mannose; yellow circle, galactose; red triangle, fucose; purple diamond, sialic acid. Reproduced with the modification and permissions from Cho et al. (2019). *p < 0.05; **p < 0.01; and ***p < 0.001.

Recently, Cho et al. (2019) applied this C18-LC-MS platform to investigate permethylated N-glycan expressions in AD cerebrospinal fluid (CSF). Over 88 glycans were identified and quantified in AD patient CSF and healthy controls. Several glycan extracted ion chromatograms (EIC) among different AD and control groups were demonstrated to show the separation of glycans and their differential abundances, as shown in Figure 4A. Further investigation revealed multiple abundant glycans that significantly altered between AD and controls either in female CSF (Figure 4Bi) or male CSF (Figure 4Bii). These glycans exhibited high AUC in the ROC analysis, as shown in Figures 4Biii,iv. The approach in this study also ensured the minimal sample volume (10 μL) of CSF relative to previous studies, making it a promising method of CSF glycomic neurological analysis. In addition to a conventional C18 column, most recently, a micro pillar array based C18 column (μPAC, PharmaFluidics) has been estimated to be reliable for glycomic analysis (Cho et al., 2021b). Although μPAC has not yet been used in CNS diseases, it has potential applications in this field.

Previously, C18 material was considered unsuitable for efficient separation of glycan isomers. However, a study has reported separation using C18-LC-MS for 2-AA labeled N-glycans and aminoxyTMT labeled N-glycans in AD (Gaunitz et al., 2020). Most recently, the isomeric separation ability of C18 was demonstrated using a 200 cm long μPAC column (PharmaFluidics) (Cho et al., 2021b) for the analysis of N-glycan isomers released from coronavirus (Cho et al., 2021a). Although this technique has not been used for neurological glycomic analysis, it can be an alternative to other isomeric glycomic approaches such as PGC- or HILIC-LC-MS.

##### Porous graphitic carbon in neurological glycomics

As the main interactions on PGC are hydrophobic and polar interactions, both of which fit for permethylated glycans, PGC-LC-MS is considered a method for the separation of permethylated glycans, especially their isomers. An early study showed only partial separation of permethylated Man7 and Man8 isomers derived from RNase B (Stavenhagen et al., 2015). Then, Zhou et al., 2017a,b introduced a high temperature PGC-LC-MS method that enables an efficient isomeric separation of both permethylated N-glycans and smaller glycans such as O-glycans, ganglioside glycans, and free oligosaccharide (Cho et al., 2020). With the development of these methods, PGC-LC-MS of isomeric permethylated glycomics has been applied to address many biological issues such as breast cancer (Peng et al., 2019), liver cancer (Huang et al., 2017b), colorectal cancer (Madunić et al., 2021), and more (Huang et al., 2017b; Xu et al., 2020; Zhang et al., 2020b; Flynn et al., 2021). In addition, the use of GUI has also been estimated for PGC-LC-MS analysis of permethylated glycan isomers (Gautam et al., 2020).

**FIGURE 5:**
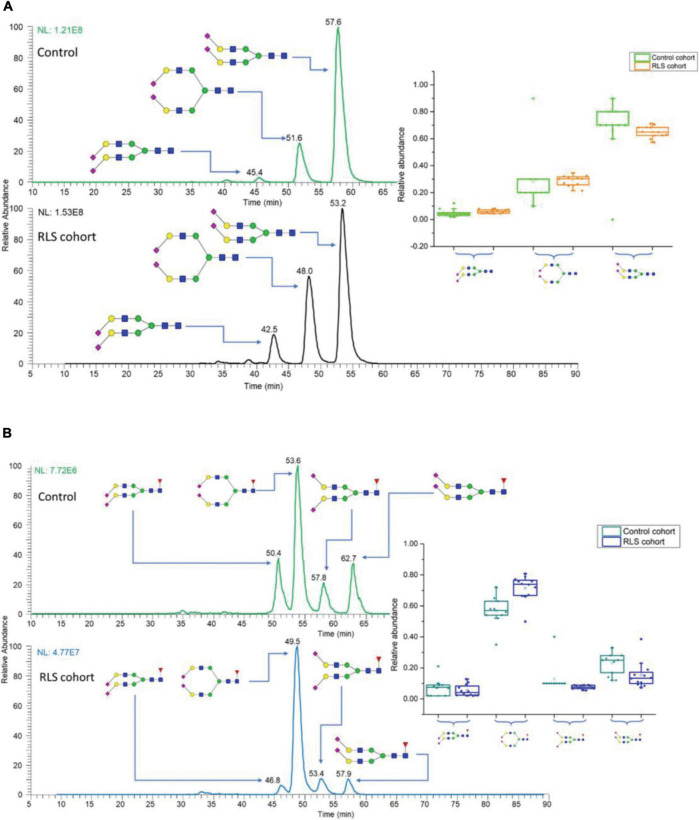
EICs of N-glycan isomers with different linkages in control and restless legs syndrome (RLS) cohorts, separated on PGC **(A)** disialylated linkage isomers, **(B)** disialylated linkage isomers with core fucose connections. Insets are the box plots showing the distributions of disialylated isomers among 10 studied subjects in the control and 12 subjects in the RLS cohorts. The box stands for the first quartile and third quartile; the line represents median; and the whiskers above and below the box set the limits for outliers. Reprinted with the permission from Dong et al. (2020).

In the field of neurological glycomics, the established PGC-LC-MS platform has been applied to characterize glycan isomers in several studies. Dong et al. (2018) investigated the isomeric distribution of permethylated glycans in an idiopathic rapid eye movement sleep behavior disorder (IRBD, considered an early sign of neurodegeneration; Assogna et al., 2021) using PGC-LC-MS (Hypercarb column, Thermo Fisher Scientific). Linkage isomers as well as positional isomers were resolved, allowing 34 glycan isomers to be identified and quantified, of which 10 isomers were significantly changed in RBD patients. Recently, the same approach was used to investigate the isomeric glycan distribution alterations from patients with restless legs syndrome (RLS) (Dong et al., 2020). A total of 38 isomers were identified, revealing 13 isomers with expression alterations in RLS patients. Figure 5 shows two examples of isomeric separation of disialylated glycans. Baseline separations for all isomers were achieved, enabling the differentiation of significant expression alterations for each isomer between RLS and control samples (insets of Figure 5).

Recently, a mesoporous graphitic carbon (MGC) column was reported to replace the PGC column (Gautam et al., 2021). The mechanism of MGC is considered similar to PGC, but the smaller particle size and pore size of MGC provides a larger surface area for a better interaction between stationary phase and glycans in LC-MS analysis. These features enabled efficient isomeric separation of permethylated glycans on a 1 cm short MGC column with higher efficiency and reproducibility than a Hypercarb nano PGC column (Gautam et al., 2021). Although this technique has not yet been employed with neurological samples, it could be a better alternative to PGC-LC-MS for isomeric glycomic analysis in CNS diseases.

### Capillary electrophoresis-mass spectrometry in neurological glycomics

Capillary electrophoresis (CE) is another major separation technique in addition to LC, providing benefits like low sample and solvent consumption and high sensitivity. Different from LC, the mechanism of CE separation is based on charges and hydrodynamic radius (sizes) of molecules (Stolz et al., 2019). Usually, glycans are required to be derivatized prior to CE-MS analysis for a complete separation. APTS through reductive amination is the routine glycan labeling method in CE-MS (Guttman et al., 1996; Guttman, 1997; Chen and Evangelista, 1998; Szabo et al., 2010). Additionally, aminoxyTMT labeling was reported to be used in CE-MS analysis of glycans (Zhong et al., 2015; Khatri et al., 2017). To date, several of the most common CE techniques employed in glycomic analysis include capillary zone electrophoresis (CZE) (Huang et al., 2017a; Lageveen-Kammeijer et al., 2019), microfluidics capillary electrophoresis (MCE) (Khatri et al., 2017; Snyder et al., 2017; Song et al., 2019), capillary gel electrophoresis (CGE), or DNA analyzer (Reusch et al., 2014; Feng et al., 2017). Because of high separation resolution, most of these CE-MS works reported isomeric separation of glycans, including resolving sialic acid linkage isomers and positional isomers. The selection of CE types were based on different analytical purposes: CZE, the most popular type, provides ultrahigh resolution (a million plates) and very low sample injection volume (nanoliters); MCE, an alternative to CZE, provides faster speed, higher resolution, lower sample volumes, and multiple functions on a single microchip (Fu et al., 2007); DNA analyzer is versatile in addition to analyzing DNAs/RNAs. These features enables the application of CE-MS in neurological glycomic analysis.

Recently, Váradi et al. (2019) introduced a CZE-MS method to monitor 2-AA labeled N-glycans derived from PD patients. A dynamic capillary coating strategy was used to allow the characterization of N-glycans using an Agilent 7100 capillary electrophoresis system coupled to an Agilent 6520 Accurate-Mass Q-TOF MS (Agilent) with the assistance of multiple stage exoglycosidases digestion. The CE-MS approach revealed 15 N-glycans whose expressions were significantly altered in PD samples when compared to controls, demonstrating the successful use of CE-MS in neurological glycomic studies. In this study, only ∼10 nL sample was injected. The smaller sample amount required by CE-MS than LC-MS could be an advantage when analyzing samples with limited quantities. However, very few CE-MS works have been focused on glycomic analysis in CNS diseases. This may be because the ultralow flow rate and necessity of salts in the migration solution can reduce the ionization efficiency of glycans, thus decreasing the sensitivity. Nevertheless, CE-MS is still a promising alternative for neurological glycomics, and should be considered in future studies.

### Ion mobility-mass spectrometry in neurological glycomics

In recent decades, a gas-phase based separation technique, ion mobility (IM), has attracted great interest from glyco-scientists. Unlike the previously described techniques, IM separation happens post-glycan ionization where ions are separated in a gas phase. In IM, ions travel in a drift cell driven by an electric field with different velocities according to their different collision cross-sections (CCS) (Baker et al., 2007). The specific CCS that is converted from drift time is related to the size and shape of an ion and is independent from the instrument parameters, where small ions travel faster than larger ions. There are several common types of IM, including drift tube ion mobility (DTIMS), traveling wave ion mobility (TWIMS), field asymmetry ion mobility or differential mobility spectrometry (FAIMS or DMS), and structures for lossless ion manipulation (SLIM) (Deng et al., 2016, 2017; Kalenius et al., 2019).

The first IM-MS study on carbohydrates, reported by Liu and Clemmer (1997), used a home-made DTIMS. Later, the same group investigated glycan profiles from esophageal adenocarcinoma serum using IM-MS, which permitted the identification of more than 20 glycans (Gaye et al., 2012). In addition to glycan profiling, IM-MS has been demonstrated to separate glycan isomers (Manz et al., 2019; Pathak et al., 2020). To enhance the separation of glycans and glycan isomers, many studies have been conducted including the use of TWIMS (Pagel and Harvey, 2013; Harvey et al., 2018), DMS (Lane et al., 2019), and SLIM (Nagy et al., 2018; Ben Faleh et al., 2019; Warnke et al., 2019; Bansal et al., 2020; Wei et al., 2020b; Yalovenko et al., 2020), the establishment of a CCS database for glycans (Struwe et al., 2016; Glaskin et al., 2017), and the assessment of glycan-metal ions (Huang and Dodds, 2013; Xie et al., 2020). Through these endeavors, IM-MS has proved to be able to perform glycan and isomer analysis. However, most of the existing IM-MS studies were only focused on glycan standards or glycans released from purified glycoproteins. There are only a few projects that involved complex biological samples (Gaye et al., 2012; Hofmann et al., 2017; Jin et al., 2019), with barely any studies related to CNS diseases. Although IM-MS provides the advantage of being much less time-consuming, it is hindered by low resolution as well as low sensitivity due to the competitive ionization. However, IM-MS provides a secondary separation dimension of glycans, making it an ideal complement to other separation techniques. The combination of prior-ionization separation and IM-MS has been demonstrated (Jooß et al., 2019) and can be a future direction for glycomic analysis of CNS diseases.

### Integration of neurological glycomics and proteomics

As discussed above, different technologies are paving a better way to identify novel glycan markers. Meanwhile, efforts have been made to combine different omic levels, such as glycomics and proteomics, to acquire a deeper understanding of biomolecular mechanisms of CNS disease progress. Wildburger et al. (2015) performed transcriptomics and glycomics to study the in-depth relations of biosynthesis pathways and glycan expressions in glioma using a glioma stem cell xenograft (GSCX) model of glioblastoma, where enrichment of high-mannose glycan biosynthesis and high-mannose glycan levels were related. Recently, the Zaia group introduced a serial on-slide tissue enzyme digestion method to achieve glycomics and proteomics with higher depth and throughput (Raghunathan et al., 2019). This method was then applied to investigate glycomic and proteomic alteration in human brain glioblastoma tissues (Sethi et al., 2022). The alteration of glycans, peptides, and glycopeptides were observed in glioblastoma samples when compared to controls. These works are examples to demonstrate the potential of integrating multiple omics for a deeper insight of neurological diseases in future studies. However, most of multi-omics studies were significantly limited by the sample size. In addition, comprehensive correlation between different omic levels is still a challenge.

## Mass spectrometry-based fragmentation techniques facilitate glycomics studies in neurological diseases

One of the main advantages offered by MS-based glycomic analysis is the acquisition of rich structural information of glycans and isomers for a better characterization. However, the structural identification remains a challenge due to the large microheterogeneity of glycosylation. The assignment of all glycans in a glycomic study has never been completely achieved. Although NMR would allow accurate structural elucidation, the fact that NMR requires lots of pure sample (usually mg scale), which is not applicable for most biological samples, has hindered its use in glycomic analysis. In addition, the lack of glycan and glycan isomer standards necessitates the development of standard independent structural identification techniques from MS.

### Characterize neurological glycans by different dissociation techniques

A variety of MS dissociation techniques have been developed to improve the identification of complex glycan structures. The deciphering of glycan structures is complicated by their microheterogeneities, particularly in the cases of glycan isomers that have lots of possible combinations of different monosaccharide linkages and positions. Therefore, different dissociation techniques are considered powerful tools to characterize the complex glycan structures and can provide compositional information through multiple glycan fragments or linkage information through cross-ring fragmentation.

#### Collision-induced dissociation/higher energy collision dissociation

The most common dissociation methods employed in glycomic studies are collision-induced dissociation (CID), collisional activation dissociation (CAD), and higher energy collision dissociation (HCD). CID happens in an ion trap, where the trapped ions are fragmented by applying an extra activation voltage, producing mostly B and Y ions (Han and Costello, 2013). CID is considered a soft fragmentation technique that mainly forms the rupture on a glycosidic bond but barely forms a cross-ring fragment. However, some derivatizations such as permethylation have provided more isomeric information in CAD (Shajahan et al., 2017). CID has been widely used in many glycomic analyses to acquire structural information for glycan compositions (Bruggink et al., 2010; Zhu et al., 2014; Everest-Dass et al., 2016; Ju et al., 2016; Gautam et al., 2020). As a common dissociation technique, CID was also applied in multiple glycomic studies for CNS diseases (Kizuka et al., 2015; Cho et al., 2019; Lee et al., 2020). In addition to CID, HCD is another common dissociation technique used in glycomic analysis. HCD happens in a HCD cell, where ions are fragmented and sent back to Orbitrap for a high-resolution analysis. Thus, compared to CID, HCD has the advantage of higher resolution and no limit of low-mass cutoff. Similar to CID, HCD has also been used for glycomic analysis of biological samples (Zhou et al., 2017b), as well as with CNS diseases (Hu et al., 2015; Wildburger et al., 2015; Kizuka et al., 2016). However, a comparison between CID and HCD indicates that CID takes advantages over HCD in studies that need a faster scan speed (Jedrychowski et al., 2011) such as proteomics for complex biological samples. While HCD commonly provided higher software identification scores and numbers, in some cases (e.g., a phosphoproteome study), CID offered larger data sets. Moreover, the stepped-collision option of HCD in modern MS instruments (e.g., Q-Exactive HF) enables a better fragmentation strategy for omics studies. In addition, the combination of HCD and IT detection can have an even faster scan speed with advantages of both methods, as was recently proved by Tu et al. (2016). However, most of the N- and O-glycomics in CNS disease studies use routine CID or HCD as their fragmentation method.

**FIGURE 6:**
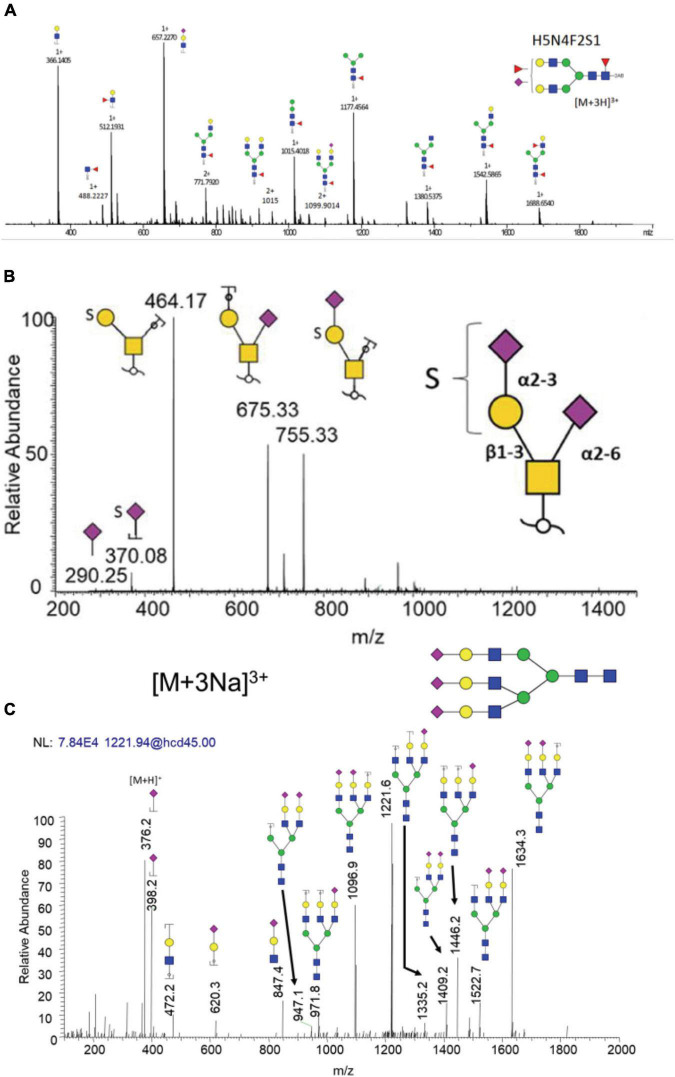
Examples of common dissociation methods used in CNS diseases. **(A)** CID of bi-antennary sialylated and fucosylated N-glycan derived from IgG of PD patients. **(B)** CID of sulfated O-glycan derived from nigrostriatal tissue of PD patients. **(C)** HCD of tri-antennary tri-sialylated N-glycan derived from neuroblastoma cell line. Reproduced with the modification and permissions of panel **(A)** from Russell et al. (2017), panel **(B)** from Wilkinson et al. (2021), and panel **(C)** from Hu et al. (2015).

Examples of common dissociation methods used in CNS diseases are shown in Figure 6. The bi-antennary sialylated N-glycan structure derived from IgG of PD patient (Figure 6A) and sulfated O-glycan structure derived from nigrostriatal tissue of PD patient (Figure 6B) are confirmed by corresponding CID MS^2^ spectra with high abundant fragment peaks assigned. Comparably, a tri-antennary tri-sialylated glycan structure is also efficiently identified by HCD MS^2^ spectrum (Figure 6C). It is easier with HCD to acquire oxonium ions for large structures than CID due to the one third *m/z* cutoff in CID. Noticeably, in all these MS2 spectra, there are no cross-ring fragmentations assigned, indicating that CID/HCD are not ideal dissociating techniques for glycan isomer identification. Therefore, there is a need for the development of novel dissociation methods to obtain adequate glycosidic bond fragmentation as well as enough cross-ring fragmentation.

#### Dissociation techniques that enhance isomeric identification

Several advanced dissociation techniques have been developed recently. Electronic excitation dissociation (EED), a high energy dissociation method, was first introduced by Yu et al. (2013). In EED, the detachment and recapture of an electron forms a di-radical that generates extensive fragments including cross-ring fragments. The optimized EED has been used to identify glycan isomers (Tang et al., 2018a,b; Wei et al., 2020a,b). An alternative to EED is ultraviolet photodissociation (UVPD), which provides selective cross-ring fragmentation (Devakumar et al., 2007). UVPD happens in an ion trap or HCD cell, where a laser beam triggers the fragmentation, providing a variety of cross-ring fragmentations. It can be combined with HCD or CID to obtain more structural information of glycans (Klein et al., 2019b). UVPD for glycomic analysis has been reported in several studies (Madsen et al., 2013; Ropartz et al., 2014, 2015, 2016; Klein et al., 2019a). Recently, a 266 nm UVPD initiated radical-directed dissociation (RDD) was introduced to enhance the glycan isomeric identification (Riggs et al., 2018) while a hybrid UVPD-CID approach, activated-electron photodetachment (a-EPD), was developed to acquire more linkage information with a less-congested spectrum (Crittenden et al., 2019).

Besides UVPD, infrared multiphoton dissociation (IRMPD) is another laser-induced fragmentation method which uses an infrared laser instead of a UV laser. Unlike EED and UVPD, IRMPD is a low energy dissociation method (Brodbelt, 2014). Therefore, its fragmentation pattern is similar to CID, but faster in analysis with less ion loss (An and Lebrilla, 2011). IRMPD has been successfully applied in glycan profiling and isomeric studies (Zhou and Håkansson, 2013; Jang et al., 2015; Tan and Polfer, 2015; Renois-Predelus et al., 2018; Schindler et al., 2018). Although these new dissociation methods have demonstrated their potential in glycomic studies, most them were insufficient in biological sample analysis, and have not been applied to address real biomedical issues. Nevertheless, the techniques offer great potential for neurological glycomics in future studies.

### Other dissociation-based techniques

#### Characterize neurological glycans by MS^n^

The MS^n^ technique is considered a powerful tool for isomeric identification of glycans. Theoretically, with consequential fragmentation it can decipher all linkage and positional information of glycan structures. However, the fact that each fragmentation round induces a dramatic decrease of signal intensity limits its application in biological samples, which rarely have enough glycan amount to go deeper than MS^3^. However, MS^3to 5^ has been employed in research to distinguish glycan isomers (Ashline et al., 2014, 2017; Kurz et al., 2021). Unfortunately, MS^n^ has not been used to address glycan alterations in CNS diseases, probably due to the difficulty of obtaining large quantities of clinical samples.

#### Targeted neurological glycan analysis by multiple reaction monitoring and parallel reaction monitoring

Besides identification techniques, targeted analysis such as multiple reaction monitoring/selected reaction monitoring (MRM/SRM) and parallel reaction monitoring (PRM) have been introduced to improve the glycan quantitation. MRM/SRM happens in a triple quadrupole MS instrument, which allows the precursor ion selection in the first quad, CID fragmentation in the second quad, and fragment ion selections in the third quad prior to the detector. Through these steps, most contaminants that have different *m/z* of either precursor or fragment ion would be removed, thus increasing the quantitation sensitivity and accuracy. An alternative to MRM, PRM is coupled to an Orbitrap instrument which provides high resolution to fragment ions. Another advantage of PRM is that it enables the monitoring of all fragment ions simultaneously, thus allowing the selection of any fragments for quantitation. The MRM for glycomic analysis has been demonstrated (Zhou et al., 2015), while PRM has been proved in a glycoproteomic study (Gutierrez Reyes et al., 2021). The pitfall of both MRM and PRM is that they cannot be utilized in untargeted glycomic analysis, thus limiting their use in initial glycomic studies of CNS diseases. However, with ever increasing glycomic studies being performed, more glycan targets in CNS diseases will be targeted, which could eventually provide a large stage for MRM and PRM in this field.

#### Analysis of neurological glycans by negative mode mass spectrometry

Most of MS-based glycomic studies for neurological glycan analysis are based on positive mode. However, several studies have demonstrated that negative ionization mode and subsequent CID or HCD fragmentation can be used to analyze glycans (Chai et al., 2001; Pfenninger et al., 2002), and provide benefit in some glycomic areas such as sulfation and sialylation which introduce negative charges to glycan structures (Kuo and Khoo, 2020). In addition, compared to positive mode (B- and Y-ions), negative mode with CID can generate informative A-type cross ring fragments in addition to C-ions, which could provide more structural information than the corresponding positive mode (Harvey, 2005a,b,c). Negative mode with HCD generates C- and Z-ions and cross-ring fragments (Szabo et al., 2017). These features of negative mode glycomics helped to identify N- and O-glycan isomers using a PGC-LC coupled to a negative-ESI-CID-MS/MS (Ashwood et al., 2018). Negative mode glycomics have also been applied to investigate bisecting glycan modifications of the β-site amyloid precursor protein cleaving enzyme-1 in Alzheimer’s disease (Kizuka et al., 2015).

## Glycan markers in nervous system diseases

### Glycan markers in Alzheimer’s disease and other dementias

Considering that most of the main proteins that have been linked to Alzheimer’s disease are glycosylated and/or are responsible for the post-translational glycosylation of other downstream proteins, the premise of a link between glycans and this burdening neurodegenerative disease is to be supported (Haukedal and Freude, 2020). Several studies have investigated the glycosylation profile of such key proteins in AD patients and discovered an abnormality in the glycan profile of AD patients: the highly expressed special N-glycans, bisecting GlcNAc, were found to be more abundant in AD patients with additional reports revealing that such an increase delays β-site amyloid precursor protein-cleaving enzyme-1 (BACE1) degradation (Hwang et al., 2010) and hence promotes AD pathogenesis (Kizuka et al., 2017). Also, the glycosyltransferase N-acetylglucosaminyltransferase III (GnT-III) which is essential for bisecting GlcNAc residue synthesis is upregulated in Aβ-treated neuroblastoma cells (Akasaka-Manya et al., 2010). In the following section, we will briefly discuss the glycosylation patterns of some that were studied over the years in relation to AD pathogenesis. In the last decades, many studies have revealed the correlation between differential glycosylation and the accumulation of the β-amyloid peptide (Aβ) within the brain (Yu et al., 2000; Kitazume et al., 2001; Yang et al., 2002; Vanoni et al., 2008; Jacobsen and Iverfeldt, 2011; Zhang et al., 2011; Kizuka et al., 2015, 2017; Yang and Qian, 2017). Although the hypothesis of Aβ plays a role in AD progress is recently no longer as attractive as before, we are summarizing some of the reported studies on Aβ.

Other studies have investigated amyloid precursor protein (APP) and its glycosylation pattern and specificity in AD considering that one of the big hallmarks of this multifactorial disease is the accumulation of the β-amyloid peptide (Aβ) within the brain (Zhang et al., 2011), Aβ being the end product of sequential cleavages of APP that occur in the amyloidogenic pathway. APP is characterized by the presence of both N-glycosylation and O-glycosylation sites which encouraged researchers to investigate the possible link between APP N-glycans and O-glycans and the production of Aβ. When Kizuka et al. (2017) manipulated glycan structures, APP transport and trafficking were altered; also, a decrease in N-glycans resulting from a disruption of their formation or maturation seems to cause a decrease in APP levels. Moreover, *in vitro* studies have revealed the role that O-GlcNAcylation plays in APP processing and hence Aβ secretion by modifying α-secretase activity (Jacobsen and Iverfeldt, 2011). Convergent results were also found *in vivo*: an increase in O-GlcNAcylation as a result of O-GlcNAcase inhibition decreases the activity of γ-secretase in 5xFAD mice (Yang and Qian, 2017). The later cleaving enzyme is composed of four subunits with only one of them known to be N-glycosylated: nicastrin. This subunit has in fact around 15 identified glycosylation sites and can be found either in its mature form when conjugated with complex N-glycans, or its immature form when complex glycosylation is absent (Yu et al., 2000). It is important to note that the glycosylation of nicastrin is reliant on the two presenilin catalytic subunits of γ-secretase (PSEN1 and PSEN2) and, considering its involvement in the substrate interactions of the cleaving enzyme (Yang et al., 2002), analyzing glycan structures in nicastrin and their possible impact on AD pathogenesis seems to be a promising objective to be worked on.

On another hand, when investigating the impact that glycosylation has on APP with the amyloidogenic pathway, studies have shown that BACE1 (or β-secretase) has four potential N-glycosylation sites which alter the folding and maturity of BACE1 hence affecting its activity and secretion rates (Vanoni et al., 2008). As previously mentioned, BACE1 in AD is characterized by the increased existence of bisecting GlcNAc on its glycosylation sites which seems to cause an increase in Aβ formation because such post-transcriptional modification causes a decrease in lysosomal degradation of BACE1 (Kizuka et al., 2015). Moreover, BACE1 activity influences post-translational modifications of other proteins such as the sialylation of its substrate β-galactoside α2,6-sialyltransferase-1 (ST6GaI1) which has been shown to increase APP and Aβ levels (Kitazume et al., 2001).

Another protein that has been of interest to Alzheimer’s disease in the field of glycosylation is the tau protein. This cytosolic enzyme, which has three N-glycosylation sites at N167, N359, and N410, has been shown to be glycosylated in AD brain, which is unexpected considering that the endoplasmic reticulum (ER) and Golgi are the sites of N-glycosylation (Sato et al., 2001). Studies have revealed the existence of three states of tau in AD brain, each representing a different stage of tau pathology of AD: AD-tau, hyperphosphorylated-tau (AD P-tau), and Paired Helical Filaments-tau (PHF-tau). Liu et al. (2002) discovered that the non-hyperphosphorylated tau was glycosylated in AD brain while it is non-glycosylated in normal brains, and that this glycosylation happens through N-linkage mainly; this makes tau more prone to become hyperphosphorylated without affecting its biological activity directly but by facilitating its substrate-interaction with cAMP-dependent protein kinase. Converging results in mice support the *in vitro* findings: the molecular weight smears of tau in AD brains were higher and reveal hyper phosphorylation of tau at multiple sites accompanied with a loss of the N-terminal portion (Li et al., 2019). Additional research has revealed that N-glycosylation at the position N359 and the absence of N-glycans at the position N410 seems to aggravate AD symptoms both *in vitro* and *in vivo* by intensifying the aggregation of tau (Losev et al., 2021).

Another protein that has appealed to researchers investigating the role of N-glycans in neurodegenerative diseases is the enzyme responsible for the degradation of the neurotransmitter acetylcholine, acetylcholinesterase (AChE). Studies revealed that the level and distribution of AChE isoforms are different in AD: in the frontal cortex of AD patients where amyloid aggregates are usually found, an elevated level of abnormally glycosylated AChE isoforms was found, which was not the case in the cerebellum where amyloid accumulation is absent (Appleyard and McDonald, 1992; Saez-Valero et al., 1999).

The brain chondroitin sulfate-glycosaminoglycan (CS-GAG) sulfation in perineuronal nets (PNNs) has also been related to AD (Miyata et al., 2012). The PNN sulfation change is more likely to promote AD pathology rather than PNN abundance (Huynh et al., 2019; Logsdon et al., 2022). Recently, Logsdon et al. (2022) discovered that CS-GAG were hyper sulfated in AD brain while the total amount of CS-GAG did not change when compared to controls using LC-MS/MS + MRM. These changes were also found to be positively related to P-tau accumulation, and further correlated with impaired cognitive function. This work highlighted the importance of CS-GAG investigation in CNS diseases and provided more clues for future studies. However, these findings might be affected by pre-mortem drug treatment, and could not explain the changes of brain matrix glycans that occurred between Mini-Mental State Examination testing and time of death.

Another indicator that commonly occurs in CNS diseases and injuries is the glial scar (Nicaise et al., 2022). Recent studies have related glial scars to neurodegenerative disease including AD (D’Ambrosi and Apolloni, 2020). Glycosylation changes have been observed and linked to spinal cord regeneration with a decrease of complex glycans in spinal cord injury models (Ronan et al., 2022). In addition, the sulfation pattern of glycosaminoglycans was also observed to be altered in active gliosis in animal models after brain injuries (Alonge et al., 2021). These investigations indicate that glycosylation and glycosaminoglycan sulfation patterns in glial scarring might be explored as potential markers to indicate CNS diseases. However, the above-mentioned studies were not able to analyze enough human cohorts to make a solid conclusion. More research in this area related to large quantities of clinical brain samples should be considered in the future.

### Glycan markers in Parkinson’s disease and parkinsonian syndromes

Even though scientists have not been able to elucidate the mechanisms by which α-synuclein aggregates and induces toxicity, it is well established that the aggregation of this protein which causes the formation of Lewy bodies is linked with the pathogenesis of Parkinson’s disease (Conway et al., 2000). And α-synuclein, like most of the human system’s proteins, can be modified post-translation through glycation and glycosylation which will alter its activity and interaction with other macromolecules such as proteins, hormones, and neurotransmitters, hence affecting its tendency to form aggregates (Vicente Miranda et al., 2017). Padmaraju et al. (2011) demonstrated that the glycation of this neuronal protein in one of its 15 lysine residues stabilizes the formed oligomers (Krumova et al., 2011) and, hence, accelerates the aggregation of α-synuclein into oligomeric forms. Accordingly, the accumulation of α-synuclein into molten globule-like structures result from the glycation of this protein with a ribose (Chen et al., 2010b). Hence, knowing that α-synuclein aggregation, and especially its aggregation in its oligomeric forms, cause increased toxicity, studying the role of AGE-α-synuclein in relation to PD pathogenesis has become important.

Many studies using mass spectrometry and western blot analysis revealed that AGEs that are present in the brains of PD patients at the periphery of Lewy bodies, in the substantia nigra, amygdala, and cerebral cortex are intensely modified (Castellani et al., 1996; Dalfo et al., 2005; Bayarsaikhan et al., 2015). Also, AGE receptors (RAGE) expression was increased in the substantia nigra and the frontal cortex in brains characterized by early stages of Parkinsonian neuropathology. Moreover, AGEs and α-synuclein are similarly distributed in Lewy bodies when double immunofluorescence was applied on pre-parkinsonian brains. Choi and Lim, who investigated the link between AGEs and α-synuclein accumulation in a mice model of parkinsonism (MPTP-induced mice), reached similar conclusions: there was co-localization of the well-known AGEs N(ε)-(carboxymethyl)lysine (CML) and N(ε)-(carboxyethyl)lysine (CEL) and the parkinsonian protein α-synuclein. They also detected greater amounts of the oligomeric form of α-synuclein in the substantial nigra region of MPTP-brains compared to the control, non-parkinsonian brains (Choi and Lim, 2010). Moreover, α-synuclein can undergo O-GlcNAcetylation at nine different sites; depending on the site(s) where the modification(s) is/are occurring, the tendency of the acetylated protein to aggregate is altered, usually leading to a decrease in α-synuclein aggregation (Levine et al., 2019).

The triggering receptor expressed on myeloid cells 2 (TREM2) surface receptor is another PD-associated protein that has been investigated in relation to its glycosylation patterns being characterized by two N-glycosylation sites on its immunoglobulin domain. This type I membrane protein, which is even expressed on microglia, seems to have anti-inflammatory properties in various diseases (Rayaprolu et al., 2013). Treatment of TREM2 with different glycosidases revealed the presence of N-linked glycosylation with high mannose and uncovered the modification of TREM2 in the Golgi by the addition of complex oligosaccharide chains (Park et al., 2015). Also, changes in the complex N-glycans that decorate TREM2 alter the conformation and hence the trafficking of this protein, disrupting its antioxidant and protective properties in some pathogenic states.

Another protein that has been inspected in relation to PD pathogenesis the DJ-1 protein, an antioxidant protein that is coded by the PARK7 gene, the latter known to be linked to familial PD. This Parkinsonism-associated protein repairs glyoxal- and methylglyoxal-glycated proteins through its deglycase activity and hence decreases AGEs by degrading the early glycation adducts (Advedissian et al., 2016). In fact, when Advedissian et al. (2016) used small interfering RNAs to deplete DJ-1 in cell culture, an important increase in protein glycation levels was observed, demonstrating the essential role that DJ-1 deglycase plays in averting protein glycation. And because α-synuclein has been demonstrated to be a substrate for DJ-1’s deglycase activity, DJ-1 thus influences α-synuclein aggregation and affects Parkinson’s pathogenesis (Sharma et al., 2019).

Parkin, which is a component of the E3 ubiquitin ligase complex encoded by the gene PARK2, is implicated in Parkinson’s pathogenesis (Imai et al., 2000). This enzyme composed of 426 amino acids not only preserves the activity and integrity of the mitochondria through its capacity to degrade many mitophagia-related membrane proteins, but has been shown to play a role in glutamatergic transmission. One important aspect of parkin is that this protein does not interact with non-glycosylated α-synuclein but does affect α-synuclein aggregation *via* its interaction with synphilin-1; this interaction has been established both *in vivo* and *in vitro*. Such findings come to support the discovery that mutations in parkin are associated with PD symptoms and progression (Chung et al., 2001; Shimura et al., 2001).

One last protein that might be of interest in relation to PD is the Narp protein. In fact, the neuronal pentraxin II (NPTXII) gene upregulated in the substantia nigra of parkinsonian brains has been shown to be in tight association with the aggregation of α-synuclein in both the substantia nigra and cerebral cortex of PD brains (Moran et al., 2008). However, even though this protein is known to have glycosylation sites, little research has focused on its glycosylation and the effect it has on its functions. Hence, more studies are to be conducted to have a better understanding of its role in PD pathogenesis.

### Glycan markers in traumatic brain injury

TBI is a critical and increasing global and socio-economic problem (Maas et al., 2017; GBD 2016 Traumatic Brain Injury and Spinal Cord Injury Collaborators, 2019). Despite its high burden, to date, patient characterization and therapeutic decision-making remain a major challenge, and novel markers capable of providing insights into the underlying pathophysiological pathways and improving clinical phenotyping are urgently needed to positively impact patient outcomes (Mondello et al., 2020). Deciphering the astonishing diversity of glycans and their pathobiological roles can enhance our understanding of the heterogeneity and complexity of traumatic brain injury (TBI) while providing opportunities to identify novel therapeutic targets (Kobeissy et al., 2022).

Previous experimental studies have indicated the potential relevance of altered glycosylation in TBI. A comparative analysis of aspirin and clopidogrel treatment in a controlled cortical impact (CCI) model has shown that glycomic changes are indicative of response to the therapeutic interventions (predictive markers) and associated with injury (neuroinflammation) and recovery patterns (Abou-Abbass et al., 2016). More recently, Mondello et al. (2022) performed the first comprehensive profiling of the N-glycome in serial serum samples from patients with moderate to severe TBI. Using cutting-edge highly sensitive liquid chromatography–tandem mass spectroscopy (LC-MS/MS) technologies, serum glycans were analyzed and characterized, identifying a detectable glycofingerprint of TBI and two brain-specific prognostic glycomarker candidates (i.e., HexNAc_5_Hex_3_ and HexNAc_5_Hex_4_NeuAc_1_). Taken together, these findings emphasize the need for biomarker research in TBI to move into the field of glycomics, which may also be a powerful tool to unveil possible pathogenetic and pathophysiological mechanism(s) and links between TBI and neurodegenerative diseases.

### Glycan markers in other CNS diseases

Glycosylation changes have also been linked to other CNS diseases. The glycomic patterns were found to be altered in Huntington’s disease (HD) mice (Gizaw et al., 2015). The increased levels of core-fucosylated and bisection N-glycans were observed in HD brain tissues when compared to the controls. In recent studies of amyotrophic lateral sclerosis (ALS) (Edri-Brami et al., 2012; Costa et al., 2019), the increase of glycan sialylation and decrease of fucosylation were found in ALS patient sera. Subsequent glycomic analysis revealed the relation of serum IgG glycans with ALS (Edri-Brami et al., 2012). In addition, galactosylation of CSF IgG could be a potential biomarker for ALS (Costa et al., 2019). Multiple sclerosis (MS) disease is another CNS disease, and studies have provided evidence that myelin oligodendrocyte glycoprotein (MOG) specific antibodies and T-cells might contribute to MS (Khare et al., 2018; Bronge et al., 2019). In addition to these neurodegenerative diseases, glycans are also associated with several neurological disorders. Highly branched plasma N-glycan structures were reported to be positively related to major depressive disorder (MDD) (Park et al., 2018), while decreased levels of agalactosylated N-glycans and increased levels of triantennary N-glycans were found in MDD serum (Boeck et al., 2018). Moreover, increased branch-fucosylation of biantennary glycans and decreased levels of some tri- and tetra-antennary glycans were identified in attention-deficit hyperactivity disorder (ADHD) children patient plasma (Pivac et al., 2011).

These studies demonstrate the importance of glycosylation in many CNS diseases which could be potential biomarkers in the future. Glycan changes have also been observed in many other diseases, including but not limited to idiopathic REM sleep behavior disorder (iRBD) (Dong et al., 2018), restless legs syndrome (RLS) (Dong et al., 2020), schizophrenia (Stanta et al., 2010; Tucholski et al., 2013), and post-traumatic stress disorder (PTSD) (Tudor et al., 2019). Although differential glycan abundances were found in different CNS diseases, the limited sample sizes in most of the studies hindered the confident discovery of glycol-markers for these diseases. More clinical cohorts need to be investigated in future studies.

## Concluding remarks

We have discussed a variety of advanced MS-based strategies and approaches for in-depth glycomic analysis. These techniques have been successfully applied toward glycan biomarker discovery in many nervous system diseases. Among them, LC-MS with various derivatization strategies is the most common approach due to its high sensitivity, resolution, reproducibility, and robustness. CE and IM could be promising complements for LC as they provide secondary separation dimensions. In addition to separation methods, multiple dissociation and acquisition strategies have also been developed to enhance the identification of glycans, especially for glycan isomers. Together, these MS-based glycomic tools have ensured reliable and efficient analysis to discover novel glycan markers in neurobiological diseases such as Alzheimer’s disease, Parkinson’s disease, traumatic brain injury, and more. However, huge challenges remain for comprehensive characterization of glycan isomers using current technologies. More efficient derivatization and separation approaches need to be developed to achieve a complete separation of all glycan isomers. Meanwhile, effective dissociation methods should also be studied where the combination of different dissociation techniques could be one resolution. In addition to technique development, the biological applications for clinical samples derived from neurological diseases should be addressed. More biomedical and clinical cohorts need to be analyzed to discover and verify potential glycan markers for better prognosis and diagnosis of these diseases.

## Author contributions

All authors listed have made a substantial, direct, and intellectual contribution to the work, and approved it for publication.
